# Supplementary material for: Intestinal endometriotic nodules with a length greater than 2.25 cm and affecting more than 27% of the circumference are more likely to undergo segmental resection, rather than linear nodulectomy
Source: PLoS One. 2021 Apr 15;16(4):e0247654. doi: 10.1371/journal.pone.0247654 (PMC8049285; doi:10.1371/journal.pone.0247654)
Supplement: Attachment — Submitted filename: Revision PlosOne.doc [file pone.0247654.s001.doc]

**“Intestinal endometriotic nodules with a lenght greater than 2.25 cm and affecting more than 27% of the circumference are more likely to undergo segmental resection, rather than nodulectomy”**

This retrospective study investigates the efficacy of intestinal ultrasonography with bowel preparation (TVUSBP) for endometriosis mapping in evaluating intestinal endometriosis in order to choose the surgical technique (segmental resection or nodulectomy).

**Materials and methods:**

- Consent for data collection and data analyses is mandatory, even in retrospective studies.
- Is the surgeon blinded respect to TVUSBP findings? Please provide more explanation about the study design.
- What are inclusions criterias? Please indicate a better explanation of initial inclusions criterias for sample selection.
- Line 146: Please specify how the measurements were performed; please provide references about the US parameters used for nodules characterization.

**Results:**

- Why did you perform surgery in asymptomatic patients with posterior compartment DIE?
- Line 245: When did you perform the study? In 2019? If so, why the study period indicated in Materials and Methods was from 2010 to 2014?
- Please provide a table for ROC curves parameters including sensitivity, specificity, NPV and PPV for each surgical technique.
- Can we give a look to Table 1? We could not find it in the manuscript.

**Discussion:**

- Please provide limits of the study (small sample size, retrospective analysis, different surgeons skills).

Additionally, the paper requires major English language revisions.
